# A systematic genomic screen implicates nucleocytoplasmic transport and membrane growth in nuclear size control

**DOI:** 10.1371/journal.pgen.1006767

**Published:** 2017-05-18

**Authors:** Kazunori Kume, Helena Cantwell, Frank R. Neumann, Andrew W. Jones, Ambrosius P. Snijders, Paul Nurse

**Affiliations:** 1 Hiroshima Research Center for Healthy Aging, Department of Molecular Biotechnology, Graduate School of Advanced Sciences of Matter, Hiroshima University, Higashi-Hiroshima, Hiroshima, Japan; 2 Cell Cycle Laboratory, The Francis Crick Institute, London, United Kingdom; 3 Laboratory of Yeast Genetics and Cell Biology, Rockefeller University, New York, New York, United States of America; 4 Protein Analysis and Proteomics Platform, The Francis Crick Institute, London, United Kingdom; Ohio State University, UNITED STATES

## Abstract

How cells control the overall size and growth of membrane-bound organelles is an important unanswered question of cell biology. Fission yeast cells maintain a nuclear size proportional to cellular size, resulting in a constant ratio between nuclear and cellular volumes (N/C ratio). We have conducted a genome-wide visual screen of a fission yeast gene deletion collection for viable mutants altered in their N/C ratio, and have found that defects in both nucleocytoplasmic mRNA transport and lipid synthesis alter the N/C ratio. Perturbing nuclear mRNA export results in accumulation of both mRNA and protein within the nucleus, and leads to an increase in the N/C ratio which is dependent on new membrane synthesis. Disruption of lipid synthesis dysregulates nuclear membrane growth and results in an enlarged N/C ratio. We propose that both properly regulated nucleocytoplasmic transport and nuclear membrane growth are central to the control of nuclear growth and size.

## Author summary

Membrane-bound organelles are maintained at a size proportional to cell size during cell growth and division. How this is achieved is a little-understood area of cell biology. The nucleus is generally present in single copy within a cell and provides a useful model to study overall membrane-bound organelle growth and organelle size homeostasis. Previous mechanistic studies of nuclear size control have been limited to cell-free nuclear assembly systems. Here, we screened a near genome-wide fission yeast gene deletion collection for mutants exhibiting aberrant nuclear size, to identify, more systematically, components involved in nuclear size control. Roles for protein complexes previously implicated in nuclear mRNA export and membrane synthesis were identified. Molecular and genetic analysis of mRNA nuclear export gene mutant cells with enlarged nuclear size revealed that general accumulation of nuclear content, including bulk mRNA and proteins, accompanies the nuclear size increase which is dependent on new membrane synthesis. We propose that properly regulated nucleocytoplasmic transport and nuclear envelope expansion are critical for appropriate nuclear size control in growing cells.

## Introduction

Much is known about the molecular mechanisms that underpin membrane trafficking and local membrane growth in eukaryotic cells [1], but how membrane-bound organelles determine their overall growth rate and maintain an appropriate size is not well understood. The simple shape of the nucleus, and the fact that it is generally present in single copy within a cell, makes it a useful model to study overall membrane-bounded organelle growth and organelle size homeostasis. Work in algae and sea urchin embryos led Hertwig in 1903 to propose that there is a constant karyoplasmic ratio characteristic of cells [2]; since then nuclear size has been reported to correlate with cell size across a range of cell types and species [2,3]. Budding and fission yeasts exhibit a nuclear size proportional to cell size, resulting in a constant ratio of nuclear and cellular volumes (N/C ratio) [4,5]. In fission yeast the N/C ratio remains constant throughout the cell cycle, and no increase in the ratio is observed during or after S phase; even a 16-fold increase in nuclear DNA content does not affect N/C ratio [5]. These results indicate that, contrary to the generally accepted view, nuclear size is not directly determined by nuclear DNA content. Increases in ploidy do result in enlarged nuclei but this occurs indirectly, via an increase in cell volume which results in an increase in nuclear size [5]. Study of multi-nucleated cells with nuclei that are unevenly distributed throughout the cell revealed that the volume of each nucleus is proportional to that of its surrounding cytoplasm [5]. Results of an *in vitro* study of *Xenopus* egg extracts demonstrated that the available space surrounding a nucleus determines nuclear expansion rate [6], consistent with the fission yeast results. Cytoplasmic effects on nuclear size were also observed when erythrocyte nuclei injected into the cytoplasm of larger HeLa cells were found to grow in size [7]. Similarly, HeLa nuclei increased in volume when injected into the cytoplasm of *X*. *laevis* oocytes [8].

These experiments indicate that nuclear size is determined by the overall size of the cell, and that the cytoplasmic content immediately surrounding a particular nucleus is important for determining its size. However, these studies have given no insight into the molecular mechanisms that control nuclear growth and nuclear size homeostasis. An important contribution to molecular mechanism was provided by Levy and Heald [9]. These authors studied nuclear assembly around exogenous DNA added to egg extracts from two species of *Xenopus*: *X*. *laevis*, with large nuclei, and *X*. *tropicalis*, with small nuclei. A GFP-NLS (nuclear localisation signal) fusion protein was found to be accumulated at a faster rate into nuclei assembled in extracts from *X*. *laevis*. The authors concluded that nuclear transport was key to establishing the differing nuclear sizes assembled *in vitro* in the egg extracts. The transport factor Impα2 (an importin) was found to be at a higher level in *X*. *laevis* extracts than in *X*. *tropicalis* extracts whereas the transport factor Ntf2 was found to have an inverse relationship. Increasing the level of Impα2 increased the size of the *in vitro* assembled nuclei and overexpression of Impα2 increased nuclear size in embryonic cells. Addition of Lamin B3, a cargo of Impα2, to extracts also increased the size of the nuclei assembled *in vitro*. This study led the authors to propose that Lamin B3, transported by Impα2 into the nucleus, plays a key role in determining nuclear size. A further study reported that total lamin concentration, rather than the level of a specific lamin, affects the size of nuclei assembled in *X*. *laevis* egg extracts as well as in *Xenopus* embryos and mammalian tissue culture cells, though different lamin concentrations have different effects in different cell types and developmental stages, sometimes increasing the lamin level increased nuclear size and sometimes decreased it [10].

Fission yeast cells lack lamins yet display nuclear size control [5], suggesting that there are other key players in nuclear size control that have not, as yet, been identified. To identify, more systematically, other proteins involved in controlling nuclear growth and nuclear size homeostasis, we have carried out a genetic screen to identify genes that when deleted alter the N/C ratio of growing fission yeast cells, and so are candidates for playing roles in nuclear size control. Our screen has identified new factors as having roles in this control, and our studies have led us to conclude that nucleocytoplasmic transport of both RNA and protein and nuclear membrane growth contribute to the overall control of nuclear growth and nuclear size homeostasis in growing cells.

## Results

### Screen for N/C ratio mutants

**Fig 1:**
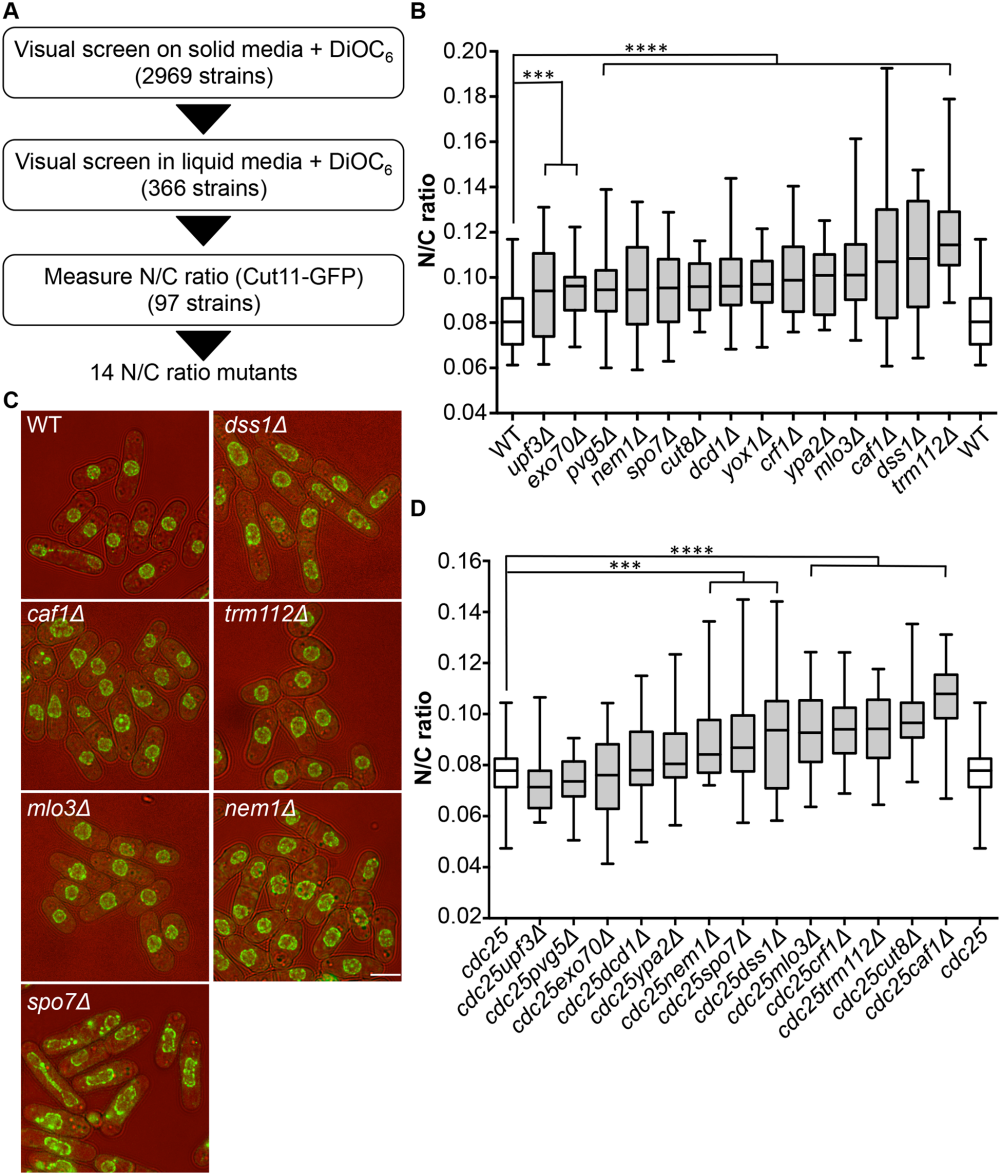
Identification of N/C ratio mutants. (a) Screen schematic. (b) N/C ratio of wild type and 14 gene deletion strains identified as having an increased N/C ratio (25°C, n>30). (c) Cell (brightfield) and nuclear envelope (Cut11-GFP) of wild type, *dss1Δ*, *caf1Δ*, *mlo3Δ*, *trm112Δ*, *nem1Δ* and *spo7Δ* cells (25°C). (d) N/C ratio of *cdc25-22* and double mutant (*cdc25-22* and candidate gene indicated) cells (36°C, 3h, n>30). *Cdc25-22yox1Δ* cells were not tested due to synthetic lethality. ***P<0.001, ****P<0.0001. Scale bar: 5 μm.

To identify genes involved in nuclear size control, we carried out a screen for fission yeast mutants exhibiting an abnormal N/C ratio. We screened a gene deletion collection consisting of 2,969 viable deletion mutants representing approximately 80% of *S*. *pombe* viable haploid gene deletion strains [11]. The screen was carried out in three stages (Fig 1A). A visual screen was carried out on solid agar, examining growing cells on the edges of colonies, using the lipophilic fluorescent dye DiOC_6_ [12] to visualise nuclear and cellular membranes; 366 strains were identified as potentially having N/C ratios greater or smaller than wild type. In a second stage, these potential N/C ratio mutants were visually screened with DiOC_6_ during steady state growth in liquid media; 97 potential N/C ratio mutants were retained. In a third stage a nuclear membrane marker Cut11-GFP was introduced into cells, and imaging was carried out during steady state growth in liquid media. Cut11 is a transmembrane nuclear pore complex protein orthologous to *H*. *sapiens* and *S*. *cerevisiae* NDC1. N/C ratios were determined as described previously [5]. We identified 14 gene deletion strains that displayed a N/C ratio at least 15% higher than that of wild type cells (Fig 1B, S1 Table); four of these, *mlo3*, *caf1*, *dss1* and *trm112* (Fig 1C), exhibited a N/C ratio greater than 0.100, which was more than 25% higher than the wild type value of 0.081. No strains with a N/C ratio significantly smaller than wild type were identified.

Mutants could generate aberrant N/C ratios as a consequence of asymmetric nuclear division instead of an interphase nuclear size control defect. If this were the case the N/C ratio would be expected to correct if cells grew for an extended time in interphase. We used the cell cycle mutant *cdc25-22* to arrest cells in interphase, and then measured the N/C ratio (Fig 1D, S1 Table). Eight mutants still exhibited significantly aberrant N/C ratios during interphase arrest; these eight mutants carried deletions of the *caf1*, *crf1*, *cut8*, *dss1*, *nem1*, *mlo3*, *spo7* and *trm112* genes, and included the four gene deletions noted above with the strongest phenotypes in normal exponentially growing cells (Fig 1B). In addition to an increased N/C ratio, two of the candidate mutants, *nem1Δ* and *spo7Δ*, exhibited nuclear shape defects (Fig 1C). Strikingly, this unbiased screen independently identified two components of two complexes, Dss1-Mlo3 and Nem1-Spo7, which are involved in mRNA export from the nucleus and membrane synthesis respectively [13,14].

### mRNA export and N/C ratio control

**Fig 2:**
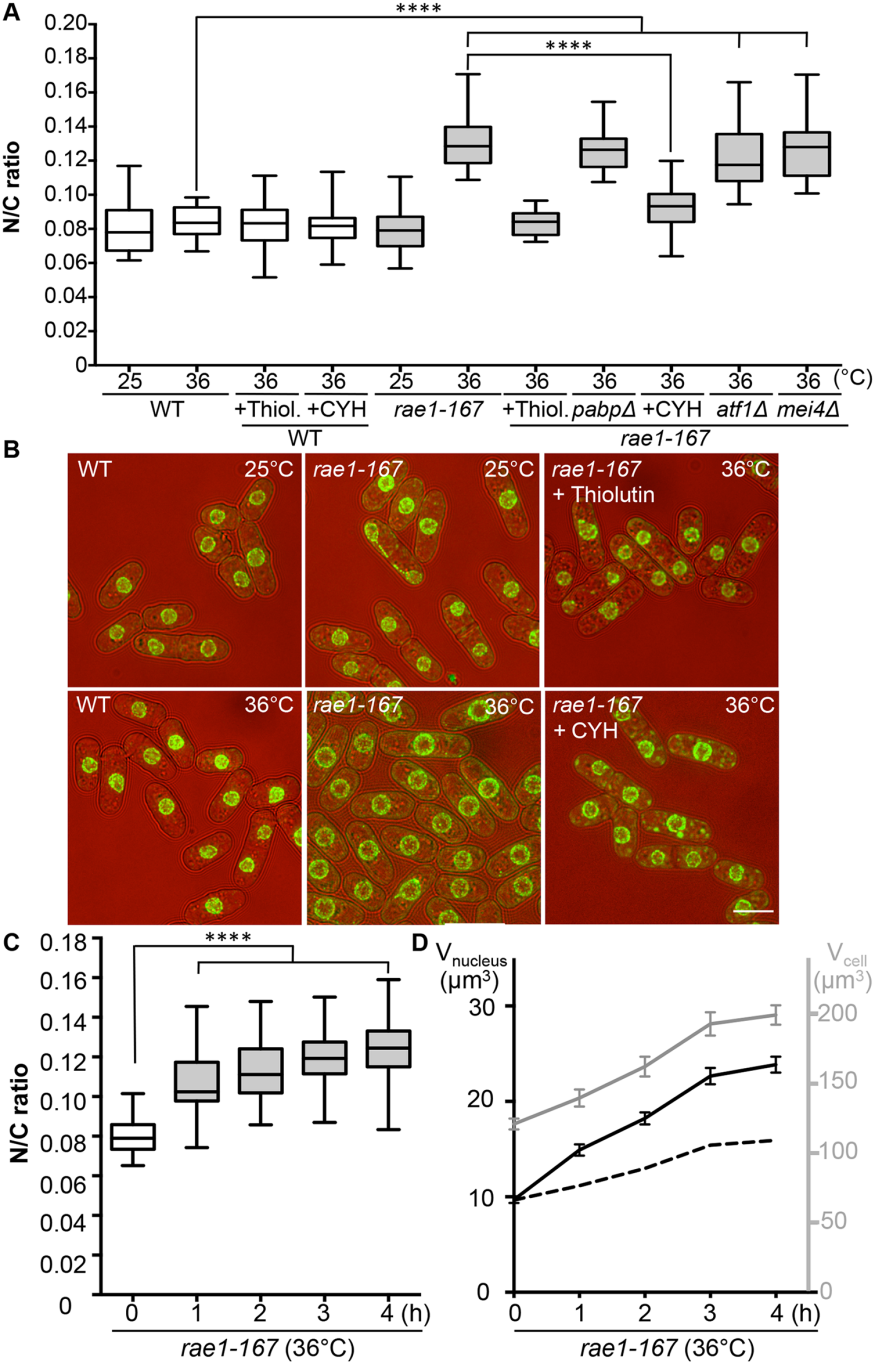
N/C ratio of *rae1-167* mutant cells. (a) N/C ratio of wild type, wild type + thiolutin (15 μg/ml) (Thiol.), wild type + cycloheximide (100 μg/ml) (CYH), *rae1-167*, *rae1-167pabpΔ*, *rae1-167atf1Δ*, *rae1-167mei4Δ*, *rae1-167* + thiolutin (15 μg/ml) (Thiol.) and *rae1-167* + cycloheximide (100 μg/ml) (CYH) cells in YE4S (25°C or 36°C, 4h, n>30). (b) Cell (brightfield) and nuclear envelope (Cut11-GFP) of indicated strains (36°C, 4h). (c) N/C ratio of *rae1-167* mutant (36°C, n>30). This phenotype is not observed in all temperature sensitive mutants; neither *cdc25-22* (Fig 1D) nor *cut6-621* (Fig 4A) temperature sensitive mutant cells show this phenotype following shift to 36°C for 4h. (d) Average cell volume (grey) and nuclear volume (black) of *rae1-167* cells (36°C). Dotted line represents nuclear volume required to maintain wild type N/C ratio of 0.08. ****P<0.0001. Scale bar: 5 μm.

The mRNA export factor Dss1 and the RNA binding protein Mlo3 (orthologous to *S*. *cerevisiae* YRA1 and *H*. *sapiens* ALYREF) are components of a complex implicated in nuclear mRNA export [13]. To investigate this further we looked at other components of the complex. A third component of the complex, Rae1, targets the Dss1-Mlo3 mRNP to the nuclear pore [13]. The *rae1* gene is essential so was not included in our screen of viable strains. To assess the N/C ratio phenotype of cells lacking Rae1 function we used the temperature-sensitive mutant *rae1-167*. *Rae1-167* cells have been reported to accumulate poly(A)+RNA in the nucleus when shifted to the restrictive temperature [15], and we confirmed that poly(A)+RNA accumulates in the nucleus after shift from 25°C to 36°C; this accumulation occurs rapidly, beginning within 15 minutes of temperature shift (S1A Fig). At 25°C, the N/C ratio of this strain was similar to that of wild type (0.080), but after incubation at 36°C for 4 hours the N/C ratio increased by more than 50% to 0.125–0.135, values greater than those of any of the viable gene deletion strains identified in our screen (Fig 2A and 2B, S2 Table). Given this more extreme effect we focused our studies on *rae1-167*. A N/C ratio increase was detectable within 1 hour of the temperature shift, and became maximal an hour later (Fig 2C). This N/C ratio increase was caused by an increased nuclear growth rate, which was approximately twice that required to maintain a constant N/C ratio (Fig 2D). To determine whether this increase in the N/C ratio caused by inhibiting mRNA export required continued RNA synthesis, we treated cells with 15 μg/ml thiolutin which completely blocks RNA synthesis in wild type cells [16,17]. Although thiolutin treatment did not affect the N/C ratio of wild type cells, the treatment suppressed the increase of N/C ratio observed in *rae1-167* cells following temperature shift (Fig 2A and 2B, S2 Table). These results indicate that defects in mRNA export can change the N/C ratio, and that this change requires continued RNA synthesis.

### Protein transport and N/C ratio control

**Fig 3:**
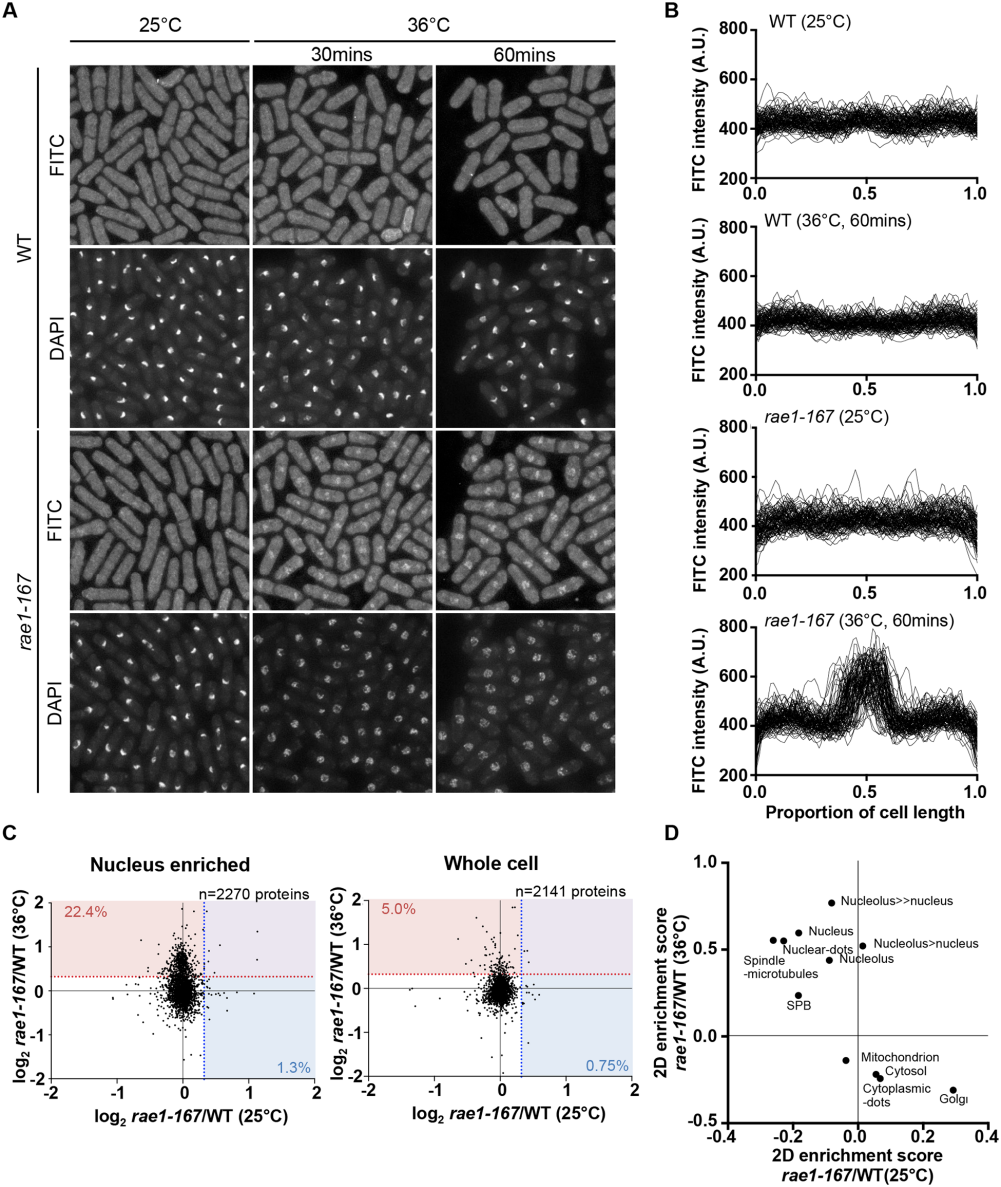
*Rae1-167* cells accumulate protein in the nucleus. (a) Protein distribution in wild type and *rae1-167* cells (25°C or 36°C, 30 mins or 60 mins) stained with FITC. (b) Quantification of FITC fluorescence distribution along the long cell axis, normalised by cell length, in wild type (WT) and *rae1-167* cells (25°C or 36°C, 60 mins, n = 100). (c) SILAC mass spectrometry analysis. log_2_ transformed *rae1*/WT ratios for individual proteins at 25°C and 36°C in nucleus enriched and whole cell samples. Dotted lines represent 25% higher protein level in *rae1-167* than WT, and values represent percentage of proteins exceeding this, at 36°C (red) and 25°C (blue). (d) 2D enrichment analysis of nucleus enriched samples in (c).

In addition to nuclear poly(A)+RNA accumulation, at 36°C *rae1-167* cells exhibit nuclear accumulation of poly(A)-binding protein (Pabp) which shuttles between the nucleus and the cytoplasm in wild type cells (S1B Fig) [18]. Pabp-GFP accumulated in the nucleus within 1 hour of temperature shift in 100% of *rae1-167* cells (S1C and S1D Fig). However, deletion of the *pabp* gene did not significantly suppress the high N/C ratio of *rae1-167* cells, so accumulation of Pabp is not sufficient to cause the N/C ratio increase (Fig 2A, S2 Table). To assess whether proteins more generally accumulated in the nuclei of *rae1-167* cells, we used the fluorescent protein-staining dye fluorescein isothiocyanate (FITC). DAPI staining was used to identify nuclei, and nuclei of *rae1-167* cells appear to show reduced compaction of chromatin following shift to the restrictive temperature (Fig 3A). Proteins were uniformly distributed throughout nuclei and cytoplasms of both wild type and *rae1-167* cells at 25°C. Within 30 minutes of shift to 36°C, *rae1-167* cells exhibited significant nuclear protein accumulation, in contrast to temperature shifted wild type cells which still had a uniform protein distribution between the nucleus and the cytoplasm (Fig 3A and 3B). This result suggested protein accumulation following mRNA export inhibition also contributes to the N/C ratio increase observed, consistent with previous observations in which prolonged inhibition of nuclear export of proteins by Leptomycin B (LMB), an inhibitor of exportin Crm1 [19], increased the N/C ratio [5]. To investigate this possibility further we tested whether the N/C ratio increase following mRNA export inhibition requires continued protein synthesis by treating cells with 100 μg/ml cycloheximide (CYH) which inhibits protein synthesis [20]. Although CYH treatment did not affect the N/C ratio of wild type cells, the treatment largely suppressed the high N/C ratio observed in *rae1-167* cells following shift to 36°C (Fig 2A and 2B, S2 Table). This result indicates that continued protein synthesis is required for the N/C ratio increase in these cells.

### Characterisation of nuclear content in *rae1-167* cells

We next tested whether it was general bulk protein and mRNA accumulation or accumulation of a smaller number of specific proteins and mRNAs which was taking place during nuclear enlargement. We characterised the protein and mRNA content of *rae1-167* nuclei at the level of individual proteins and mRNAs using SILAC mass spectrometry and microarray analysis respectively.

To identify proteins accumulated in *rae1-167* nuclei with an enlarged N/C ratio, we compared the protein content of wild type and *rae1-167* nuclei. We enriched for nuclei to allow detection of low abundance nuclear proteins that may not be detected in whole cell samples. We confirmed that nuclear enriched samples were enriched for nuclear-localised and depleted for cytosol-localised proteins (P<0.02) (S3 Table). When we compared the protein content of nuclear enriched samples, there was an increase in a subset of proteins in *rae1-167* relative to wild type at the restrictive temperature, that was not observed at the permissive temperature. 22.4% of proteins detected at 36°C (509/2270 proteins detected), in contrast to only 1.3% at 25°C (30/2270 proteins detected), were present in the *rae1-167* nucleus enriched sample at a level at least 25% higher than in wild type (Fig 3C). Proteins found at this level at 36°C and not at 25°C are listed in S4 Table. To examine what classes of proteins were enriched in *rae1-167* nuclei we carried out 2D enrichment analysis [21]. The proteins enriched in *rae1-167* nuclei relative to wild type nuclei at 36°C and not at 25°C showed significant enrichment of proteins reported to be localised to the nucleus and subnuclear structures in *S*. *pombe* [19] (Fig 3D). Enrichment of specific gene ontology (GO) categories is shown in S2 Fig; nucleic acid binding proteins were enriched in proteins increased in *rae1-167* nuclei relative to wild type nuclei at 36°C and not at 25°C. Therefore, our analysis suggests that bulk accumulation of many different proteins localised to the nucleus is taking place in *rae1-167* nuclei when the N/C ratio is enlarged, rather than accumulation of a few specific proteins.

We next investigated the steady-state levels of individual mRNA transcripts by microarray analysis to assess whether the accumulation observed is of a few specific mRNAs or bulk accumulation of many mRNAs. Levels of 888 mRNAs were increased at least 2-fold in *rae1-167* cells at 36°C relative to 25°C (S5 Table). These included mRNA transcripts of 106 common environmental stress response (CESR) genes [22] and 64 meiotic genes [23]. To examine whether increased expression of either of these specific groups of genes causes the N/C ratio enlargement observed in *rae1-167* cells, we used the transcription factor mutants, *atf1Δ* and *mei4Δ*, which are respectively defective in induction of most CESR genes and of the middle meiotic genes [22,24]. Deletion of neither *atf1* nor *mei4* suppressed the high N/C ratio of *rae1-167* cells (Fig 2A, S2 Table), indicating that specific transcription-driven increases of CESR gene or middle meiotic gene mRNAs are not the cause of the increased N/C ratio of *rae1-167* cells. It is possible that retaining mRNAs in the nucleus could affect their stability; in this situation increased levels of these specific groups of mRNAs could be causative of the nuclear size increase because *atf1Δ* and *mei4Δ* mutants would be unlikely to affect their steady state level. Taken together, our mass spectrometry and microarray analyses indicate that general bulk accumulation of a large number of proteins and mRNAs occurs in *rae1-167* cells when an N/C ratio increase is observed, and that many of these proteins are normally localised in the nucleus.

### Membrane synthesis and N/C ratio control

**Fig 4:**
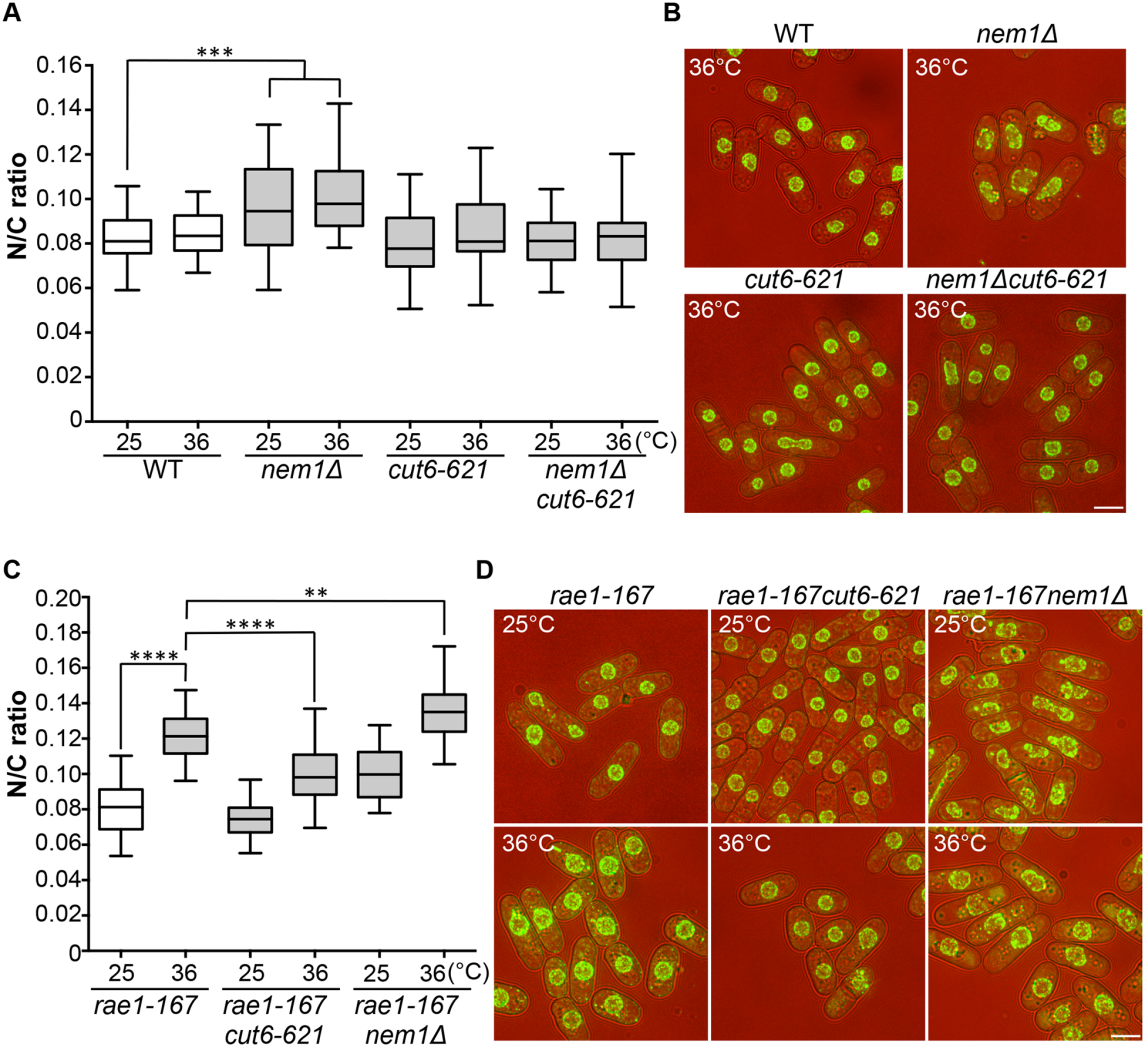
N/C ratio enlargement and nuclear membrane growth. (a) N/C ratio of wild type, *nem1Δ*, *cut6-621*, and *nem1Δcut6-621* cells (25°C, 4h 36°C, n>30). (b) Cell (brightfield) and nuclear envelope (Cut11-GFP) of the strains in (a). (c) N/C ratio of *rae1-167*, *rae1-167cut6-621* and *rae1-167nem1Δ* cells (25°C, 4h 36°C, n>30). (d) Cell (brightfield) and nuclear envelope (Cut11-GFP) of the strains in (c). **P<0.01, ***P<0.001, ****P<0.0001. Scale bar: 5 μm.

Two more enlarged N/C ratio candidates identified in our screen were *nem1* and *spo7*. These encode the catalytic and regulatory subunits of the Nem1-Spo7 phosphatase complex, responsible for dephosphorylation and activation of Ned1, a lipin family phosphatidic acid phosphatase [14,25]. In addition to the nuclear size phenotype in these mutants, nuclear shape deformation suggestive of nuclear envelope overproliferation was also observed [26]. These observations suggest that correct regulation of lipid metabolism leading to changes in membrane synthesis plays a role in nuclear size control, and we hypothesised that the increased N/C ratio of *nem1* and *spo7* deletion mutant cells may be caused by inappropriate nuclear envelope expansion. If this is the case, then inhibition of fatty acid synthesis, which is required for membrane growth [25,26,27], should suppress the increased N/C ratio observed in these mutant cells. To test this, we used a temperature-sensitive mutant *cut6-621*, which is defective in acetyl-CoA carboxylase and impaired in fatty acid metabolism [28]. The *cut6-621* mutant blocked the increase in nuclear size of *nem1Δ* cells and suppressed the nuclear shape change (Fig 4A and 4B). There was no effect of *cut6-621* on the N/C ratio of *nem1+* cells. These results indicate that *nem1* deletion leads to overproduction of phospholipid by inactivation of Ned1, causing inappropriate expansion of the nuclear envelope resulting in a N/C ratio increase. We next investigated whether new membrane synthesis is required for nuclear enlargement in the *rae1-167* mutant, by combining the *rae1-167* and *cut6-621* mutations and measuring the N/C ratio. The N/C ratio of the double mutant is significantly lower than that of the *rae1-167* mutant following shift to 36°C (Fig 4C and 4D, S2 Table), indicating that new membrane synthesis is required for the nuclear size increase observed in *rae1-167* cells.

Combining the *rae1-167* mRNA export mutant with the *nem1Δ* membrane synthesis regulation mutant led to a further N/C ratio increase, greater than that observed in either single mutant (Fig 4C and 4D). This suggests that two distinct processes are implicated in nuclear size control, membrane synthesis and nucleocytoplasmic transport. The shape phenotype of the *nem1Δ* single mutant was also suppressed by the *rae1-167* mutation (Fig 4D).

## Discussion

Our screen of viable fission yeast gene deletion strains identified 8 genes that when deleted lead to an increased N/C ratio in both exponentially growing and interphase arrested cells. Significantly, among these 8 were 4 genes encoding two components of a complex involved in nuclear mRNA export (*dss1* and *mlo3*) and two components of a complex involved in lipid metabolism (*nem1* and *spo7*). We did not find any gene deletions with a N/C ratio smaller than wild type, suggesting that reduced nuclear size might be deleterious, resulting in lethality. A screen of the diploid heterozygous gene deletion collection [11] could be expected to have less severe effects and may reveal genes that have decreased N/C ratios. It is possible that the rapid growth of unicellular fission yeast cells may require rapid ribosome biogenesis, which results in a limited range of N/C ratio perturbations being viable. A screen of essential genes may identify more severe phenotypes. The four genes, *mlo3*, *caf1*, *dss1* and *trm112*, with the strongest deletion phenotypes increased the N/C ratio by 25%, an increase that was also observed in enlarged mutant cells blocked in interphase. All four genes are thought to be involved in RNA metabolism, and two of them, *dss1* and *mlo3*, encode protein components of a complex required for nuclear mRNA export. We examined a third component of this complex, Rae1, which associates with the nuclear pore. A temperature-sensitive mutant of this essential gene, *rae1-167*, showed a 50% N/C ratio increase at the restrictive temperature, an increase greater than that seen in *dss1Δ* or *mlo3Δ* cells. The 50% N/C ratio increase was the result of a doubling of nuclear growth rate, and required continued RNA and protein synthesis. Both the mRNA and protein content of the enlarged nucleus increased. Mass spectrometry and microarray analyses showed that bulk nuclear accumulation of a large number of different proteins and mRNAs, rather than accumulation of a few specific proteins and mRNAs was taking place. This suggests that general accumulation of nuclear content contributes to the N/C ratio increase observed, though it is also possible that one, or a subset, of the many accumulated proteins and mRNAs may more specifically effect the N/C ratio increase.

Membrane growth is required for the nuclear size increase of *rae1-167* cells. The *cut6-621* mutant impairs nuclear membrane growth and also prevents the *rae1-167* nuclear size increase. The importance of membrane growth for nuclear size was also shown by our identification of two lipid metabolic genes, *nem1* and *spo7*, encoding proteins that form a phosphatase complex responsible for activation of Ned1, a lipin family phosphatidic acid phosphatase [14]. Inactivation of Ned1 by either *nem1Δ* or *spo7Δ* induced nuclear envelope expansion, resulting in aberrant nuclear shapes and an increased N/C ratio. These changes were observed in cells blocked in interphase and so are not due to aberrant mitosis. These phenotypes, like the N/C ratio increase of *rae1-167* cells, were suppressed by inhibition of nuclear membrane growth by the *cut6-621* mutation. These observations are consistent with observation that a phosphomimetic mutant of Ned1 exhibits a 34% increase in nuclear surface area in interphase [14]. The *rae1-167nem1Δ* double mutant exhibited a N/C ratio increase greater than that of either single mutant, suggesting that two distinct processes are important for nuclear size control, both bulk nucleocytoplasmic transport and nuclear membrane growth.

In addition to the components of the Dss1-Mlo3 and Nem1-Spo7 complexes, our systematic screen identified four further gene deletion strains exhibiting enlarged N/C ratios. These carried deletions in *caf1*, *cut8*, *crf1* and *trm112*. *Caf1* encodes a deadenylase of the CCR4-NOT complex. The multifunctional CCR4-NOT complex has been implicated in many different areas of gene expression, both nuclear and cytosolic. These include regulation of histone modification, regulation of transcription initiation and elongation, nuclear poly(A)-RNA degradation, mRNA export, cytosolic poly(A)-RNA decay and protein turnover [29, 30]. It is possible that disruption of one or a few of these roles of the CCR4-NOT complex by *caf1* deletion could lead to nuclear poly(A)-RNA accumulation and therefore effect nuclear size increase by the same mechanisms as those in play in *rae1-167* cells. However, due to the diverse roles this complex plays, a separate mechanism is also possible. It is possible that *cut8* deletion may cause nuclear size increase by similar mechanisms to those in *rae1-167* cells as it encodes a nuclear proteasome tethering factor [31] so could affect nuclear protein levels. *Cut8* mutant cells have also been reported to accumulate poly(A)-RNA in the nucleus at the restrictive temperature suggesting a possible role in mRNA export [32]. Although we have focused on the mRNA export role of Dss1 in this study because another component of the Dss1-Mlo3 complex was also identified by our screen, it is of note that Dss1, like Cut8, has been implicated in proteasome function [33]. *Crf1* is predicted to encode a TOR-responsive transcriptional corepressor, and deletion of its *S*. *cerevisiae* orthologue perturbs repression of ribosomal protein gene transcription [34]. The *S*. *cerevisiae* orthologue of *trm112* regulates methylation of tRNAs, rRNAs, and translation factors, and is required for synthesis of both 40S and 60S ribosomal subunits [35]. As ribosome biogenesis is dominant in rapidly growing yeast cells and involves both nuclear import of ribosomal proteins and export of ribosomal subunits, it is possible that perturbing the biogenesis process, for example by *crf1* or *trm112* deletion, could lead to the accumulation of dysfunctional ribosomes or their constituents in the nucleus thus influencing nuclear size. Therefore, the remaining candidates from our screen all encode proteins that might have roles in regulating overall RNA and protein levels, and so impact the RNA and protein content of the nucleus.

Nucleocytoplasmic transport has also been implicated in nuclear size control in metazoa. The study of nuclear assembly in *Xenopus* egg extracts discussed in the Introduction, and subsequent studies in *Xenopus* egg extracts and mammalian cells have implicated the transport factors Impα2 and Ntf2 and the import of lamins in determination of nuclear size [9,10,36]. Our data indicates that there are roles for other components involved in nucleocytoplasmic transport and also for nuclear envelope growth in nuclear size homeostasis. Our *in vivo* study of fission yeast cells in steady state growth has revealed a role for nucleocytoplasmic transport of mRNAs and proteins in interphase nuclear size control, which is dependent on continued RNA and protein synthesis.

Our work has shown that both the accumulation of nuclear content and membrane synthesis, as well as the linkage between these two processes, must be considered when proposing potential models of nuclear size control. Outlined below are two examples of the types of mechanisms for maintenance of the N/C ratio that take account of these considerations. In one, overall cytoplasmic content determines how much protein and RNA is imported into the nucleus, and as a cell grows the resulting increase in nuclear content stimulates new membrane growth, enlarging the nucleus in balance with the cytoplasm. Nuclear content could promote nuclear envelope expansion in one of two ways: increased bulk RNA and protein import could put pressure on the nuclear membrane altering its tension and inducing its expansion [37], or the increased bulk RNA and protein import could lead to increased import of one or a group of specific RNAs and proteins that bring about nuclear envelope expansion. In a second model we suggest that overall cytoplasmic content determines the growth rate of the nuclear membrane, perhaps operating through global cellular membrane growth being controlled by cell size, with a certain proportion of total cellular membrane being delivered to the nucleus. The increased nuclear surface area would result in the incorporation of more nuclear pore complexes, resulting in increased import of protein and RNA, increasing nuclear size in balance with the cytoplasm. Both mechanisms implicate nucleocytoplasmic transport and nuclear membrane growth in interphase nuclear size control, and so are compatible with the data presented here and by others elsewhere. However, in the first model it is the accumulation of nuclear content that is the initial driver of nuclear growth, whilst in the second it is growth of the nuclear membrane. Obviously these are only examples of possible mechanisms.

We conclude that appropriately regulated nucleocytoplasmic transport and nuclear membrane growth are central to nuclear size control. This may be relevant to disease states given that abnormal nuclear size and shape phenotypes are observed in many diseases [38,39] but the role of nuclear size in the pathology of these diseases remains unclear. The genetic identification of processes involved in nuclear size control in fission yeast provides a tractable system in which to investigate this control, and contributes to our understanding of how membrane-bound organelles regulate their overall growth and size.

## Materials and methods

### Yeast general methods

Strains used are listed in S6 Table. Gene tagging was performed by PCR and homologous recombination [40]. *S*. *pombe* media and methods as described previously [41]. For mass spectrometry, cells were grown in SILAC media; heavy labelled samples were grown for >8 generations in media supplemented with heavy arginine (L-Arginine:HCL (U13C6, 99%)) and heavy lysine (L-Lysine:2HCl (U13C6, 99%)) (Cambridge Isotope Laboratories Inc.). All other strains were grown in YE4S. SILAC media used was EMM (6 mM ammonium chloride) supplemented with 0.25 mg/ml leucine, 0.15 mg/ml uridine, 0.04 mg/ml arginine and 0.03 mg/ml lysine [42]. YE4S used was Yeast extract (Difco) supplemented with adenine, leucine, uracil and histidine at 225 mg/l.

### Screen for N/C ratio mutants

The 3-stage screen was carried out as described above. Firstly, deletion mutants were incubated at 25°C for 12 to 20h in 300 μl of YE4S in 96-well plates then inoculated onto YE4S agar plates containing DiOC_6_ at 10 μg/ml (Life technologies) using a pin tool (V & P Scientific, Inc) and incubated at 25°C for 12 to 20h. The N/C ratio of each mutant strain was estimated by comparison with the wild type strain growing in the same plate using a Zeiss Axioskop 40 microscope. 102 of the 2,969 deletion mutants failed to grow on plates so were excluded from the screen. 366 mutant strains were selected for a secondary visual screen in liquid medium. Cells were collected from individual exponentially growing cultures of the 366 candidate mutants, stained with DiOC_6_ and visually screened to estimate the N/C ratio. The 97 strains selected for the tertiary screen were tagged with the nuclear envelope marker protein Cut11-GFP and the nuclear and cellular volumes measured to assess the N/C ratio [5]. Images were analysed using ImageJ (NIH) as previously described [5]. Nuclear volume of shape mutants was calculated using ImageJ.

### Statistical analysis

Unpaired Student’s *t* tests were used in Figs 1B and 1D, 2A and 2C, 4A and 4C to test statistical significance in pairwise comparisons.

### Microscopy and image analysis

For visual screening, mutant cells were observed using a Zeiss Axioskop 40 microscope equipped with a 63x/1.4 NA objective and an AxioCam MRm camera. For protein and poly(A)+RNA localisation and N/C ratio measurement, cells were imaged using a DeltaVision Elite microscope (Applied Precision) comprised of an Olympus IX71 wide-field inverted fluorescence microscope, an Olympus Plan APO 60x oil, 1.42 NA objective, and a Photometrics CoolSNAP HQ2 camera (Roper Scientific). Images were captured in 0.3 or 0.4 μm z-sections over 5 μm and deconvolved using SoftWorx (Applied Precision). Projections of Cut11-GFP images were combined with bright field images. FITC intensity was measured using ImageJ (NIH).

### FITC staining

5 ml of exponentially growing cells were fixed with 70% ethanol at 4°C for at least 30 mins, washed with phosphate buffered saline (PBS) then resuspended in PBS containing 1 μg/ml fluorescein isothiocyanate (FITC) (invitrogen). 4,6-diamidino-2phenylindole (DAPI) was used to stain DNA.

### *In situ* hybridisation

The *in situ* hybridisation method used was described previously [43]. Oligo (dT)_50_ 3’-end labeled with cy3 was used as the hybridisation probe. DAPI was used to stain DNA.

### Microarrays

RNA was isolated by acid-phenol extraction and purified using RNeasy (Qiagen). Biotinylated cRNA was hybridised onto GeneChip Yeast Genome 2.0 arrays (Affymetrix) which were scanned with a GeneChip Scanner 3000 and analysed with GCOS v1.4 (Affymetrix) using default analysis settings and global scaling normalisation [44]. NCBI GEO accession number: GSE81666.

### Mass spectrometry

Exponentially growing cells were incubated at 36°C or 25°C (as indicated) for 1 hour. Wild type (heavy labeled (H)) and *rae1-167* (light labeled (L)) were mixed 1:1 by optical density (inverse labels also mixed). Whole cell and nuclear enriched protein samples were extracted.

For in-gel digestion each SILAC sample was loaded onto a NuPAGE Bis-Tris Protein Gel, 1.0 mm, 10-well (Thermo Fisher), and allowed to migrate through the gel before being stained with coomassie blue. Polyacrylamide gel slices were prepared for mass spectrometric analysis using the Janus liquid handling system (Perkin-Elmer). Each lane was excised into eight equally sized protein gel pieces, destained with 50% acetonitrile + 50 mM ammonium bicarbonate, reduced with 10 mM DTT, and alkylated with 55 mM iodoacetamide. After alkylation, the proteins were digested with 6 ng/μl trypsin overnight at 37°C. The resulting peptides were extracted in 2% formic acid/1% acetonitrile.

Samples were analysed by LC-MS/MS. An LTQ-Orbitrap Velos coupled to an UltiMate 3000 HPLC system for on-line liquid chromatographic separation was used for data acquisition. The extracted peptides were separated over a 70 min gradient elution (75 μm × 50 cm C_18_ column) with collision-induced dissociation (CID) selected as the activation method.

MaxQuant 1.3.0.5 was used for data processing and quantification. Default MaxQuant parameters were used with the following adjustments: Lys6 and Arg6 were the heavy labels, ‘Filter labelled amino acids’ was deselected, re-quantify was selected with the instruction to keep low-scoring versions of identified peptides within parameter groups and match between runs was selected. Data was searched against a UniProt extracted *S*. *pombe* FASTA file amended to include common contaminants. Normalised H/L ratios were used for analysis in Perseus 1.4.0.2. Average log_2_ *rae1-167*/WT ratios were calculated, data was annotated with ORFeome localisation data [19] and Gene Ontology terms (default Perseus GO annotation lists), and 2D enrichment analysis was carried out [21] (Benjamini-Hochberg FDR truncation threshold: 0.02). The mass spectrometry proteomics data have been deposited to the ProteomeXchange Consortium via the PRIDE [45] partner repository with the dataset identifier PXD004530.

### Protein extraction for mass spectrometry

Whole cell samples were produced by quenching cells by adding ice cold 100% (w/v) trichloroacetic acid to a final concentration of 10%. Cells were incubated on ice for at least 20 minutes, washed in ice-cold acetone and stored at -80°C. Pellets were washed and resuspended in lysis buffer (8 M urea, 50 mM ammonium bicarbonate, 5 mM EDTA and cOmplete Mini EDTA-free protease inhibitor cocktail (Roche)). 0.4 mm diameter acid washed glass beads (Sigma) were added and samples beaten to break cells (FastPrep120). Cell debris was pelleted and supernatant protein extracts stored at -80°C.

Nuclear enriched samples were produced using a protocol based on [46] and Experiment 18 [47]. Cells were harvested from 1 L cultures by centrifugation, washed in S buffer (1.4 M sorbitol, 40 mM HEPES and 0.5 mM MgCl_2_ at pH 6.5), resuspended in S buffer + 10 mM β-mercaptoethanol + 1 mM phenylmethanesulfonyl fluoride (PMSF) and incubated at 32°C for 10 minutes. Cells were harvested by centrifugation and the pellet was resuspended in S buffer + 1 mM PMSF containing 20 mg/gram cell pellet Zymolyase 100T (Amsbio) and incubated at 32°C until cell wall digestion was complete (confirmed by SDS lysis). Remaining steps were performed on ice. Cells were pelleted and washed four times in S buffer then resuspended in 20 ml F buffer (18% Ficoll 400 (w/v), 20 mM PIPES and 0.5 mM MgCl_2_) + 1 mM PMSF and lysed using a dounce homogeniser. The lysate was layered on 20 ml GF buffer (7% Ficoll (w/v), 20% glycerol, 20 mM PIPES and 0.5 mM MgCl_2_) and centrifuged at 20,000 g for 30 minutes. The pellet was resuspended in 20 ml F buffer and centrifuged at 3,000 g for 15 minutes. The supernatant was centrifuged at 20,000 g for 25 minutes. Nuclear enriched pellets were resuspended in 250 μl 2X Laemmli buffer (100 mM TRIS (pH 6.8), 4% SDS, 20% glycerol and 0.2 M dithiothreitol), heated to 99°C for 10 minutes and centrifuged. Supernatants were harvested and stored at -80°C.

Nuclear enrichment by this protocol was confirmed by preparation of whole cell and nuclear enriched samples of both wild type and *rae1-167* cells grown at 36°C (heavy and light labelled). Heavy labelled nuclear enriched sample was mixed 1:1 by protein concentration (DC Protein Assay (Bio-Rad)) with light labelled whole cell extract for each strain (inverse label mixes also produced and analysed). Samples were analysed by SILAC mass spectrometry as described. Average log_2_ nuclear enriched/whole cell ratios were calculated, data was annotated with ORFeome localisation data [19] and 2D enrichment analysis was carried out [21]. Benjamini-Hochberg FDR was used for truncation, threshold value 0.02.

## Supporting information

S1 Fig Poly(A)+RNA and poly(A)+RNA binding protein PABP-GFP localisation in *rae1-167* mutant cells. (a) Poly(A)+RNA (*in situ* hybridisation) and DNA (DAPI staining) distribution in *rae1-167* cells grown at 25°C or 36°C for 15 or 30 mins. Scale bar: 5 μm. (b) Wild type cells expressing PABP-GFP grown at 25°C or 36°C for 120 mins. (c) *Rae1-167* cells expressing PABP-GFP grown at 25°C or 36°C for 30, 60, 120, or 210 mins. Scale bars: 5 μm. (d) Frequencies of PABP-GFP distributions in *rae1-167* cells (n = 100).
(TIF)

S2 Fig GO annotated 2D enrichment analysis of nucleus enriched samples in Fig 3C. (a) Data annotated with GO biological process slim terms. (b) Data annotated with GO molecular function terms. (c) Data annotated with GO cellular component terms.
(TIF)

S1 Table Nuclear size mutants identified in genome wide screen in fission yeast. (DOCX)

S2 Table N/C ratio of indicated strains. (DOCX)

S3 Table Nucleus-localised proteins are enriched and cytosol-localised proteins depleted in nuclear enriched samples. (DOCX)

S4 Table Proteins enriched in *rae1-167* nuclear enriched samples relative to wild type (≥1.25 fold) at 36°C and not at 25°C. (XLSX)

S5 Table mRNA transcript level ratios of genes with higher mRNA transcript level (≥ 2 fold) in *rae1-167* cells at 36°C than at 25°C. (XLSX)

S6 Table Strains used in this study. (DOCX)
