# Supplementary figures and images for: A systematic genomic screen implicates nucleocytoplasmic transport and membrane growth in nuclear size control
Source: PLoS Genet. 2017 May 18;13(5):e1006767. doi: 10.1371/journal.pgen.1006767 (PMC5436639; doi:10.1371/journal.pgen.1006767)

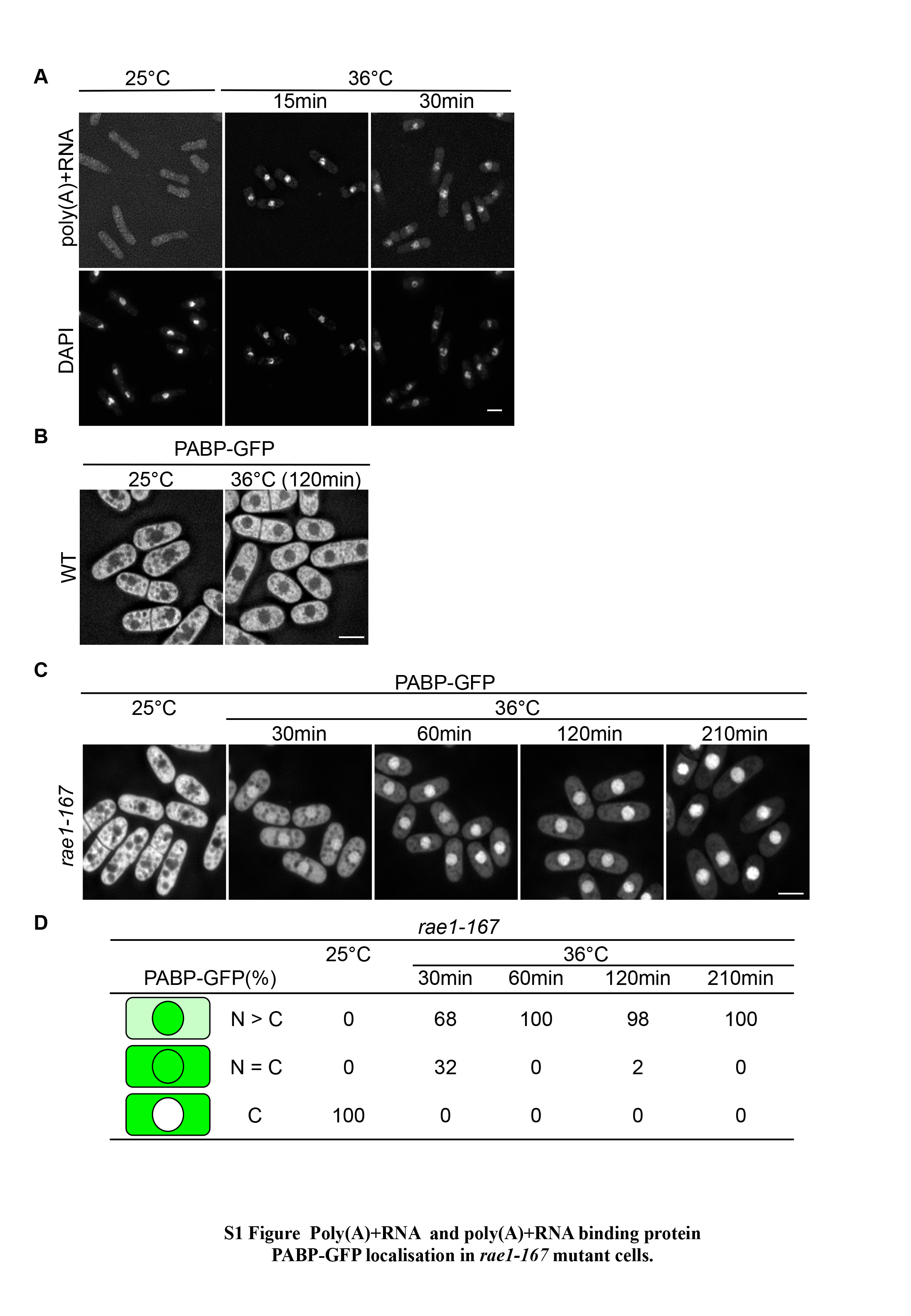

Supplement: S1 Fig — (a) Poly(A)+RNA (in situ hybridisation) and DNA (DAPI staining) distribution in rae1-167 cells grown at 25°C or 36°C for 15 or 30 mins. Scale bar: 5 μm. (b) Wild type cells expressing PABP-GFP grown at 25°C or 36°C for 120 mins. (c) Rae1-167 cells expressing PABP-GFP grown at 25°C or 36°C for 30, 60, 120, or 210 mins. Scale bars: 5 μm. (d) Frequencies of PABP-GFP distributions in rae1-167 cells (n = 100). (TIF) [file pgen.1006767.s001.tif]

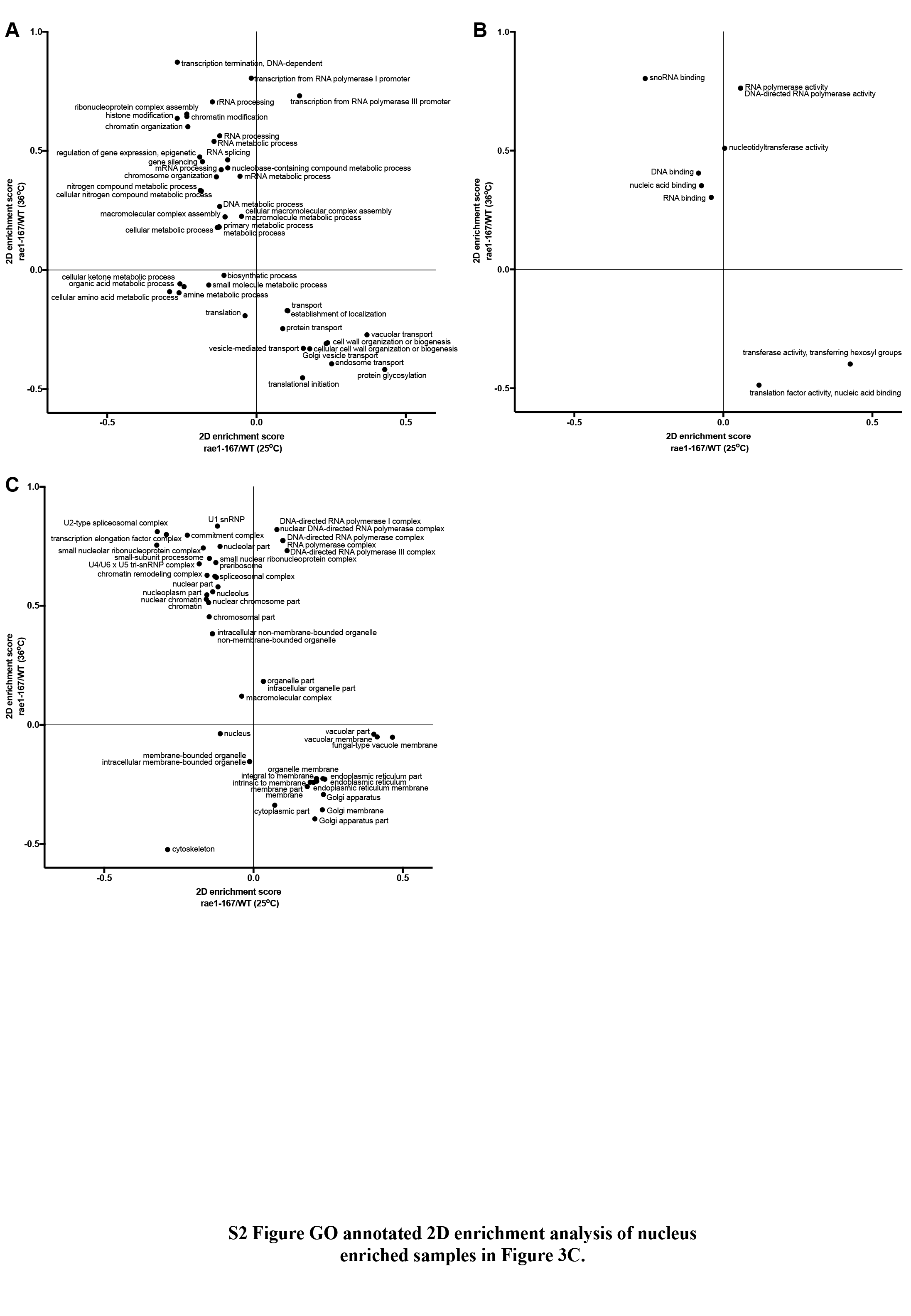

Supplement: S2 Fig — (a) Data annotated with GO biological process slim terms. (b) Data annotated with GO molecular function terms. (c) Data annotated with GO cellular component terms. (TIF) [file pgen.1006767.s002.tif]
