# Supplementary material for: A systematic genomic screen implicates nucleocytoplasmic transport and membrane growth in nuclear size control
Source: PLoS Genet. 2017 May 18;13(5):e1006767. doi: 10.1371/journal.pgen.1006767 (PMC5436639; doi:10.1371/journal.pgen.1006767)
Supplement: S1 Table — (DOCX) [file pgen.1006767.s003.docx]

|  | 25°C | | | *cdc25-22* background (36°C) | | |  |
| --- | --- | --- | --- | --- | --- | --- | --- |
| Gene  deleted | N/C ratio^a^ | Nuclear  volume^b^  (µm^3^) | Cell  volume^b^  (µm^3^) | N/C ratio^a^ | Nuclear volume^b^  (µm^3^) | Cell volume^b^  (µm^3^) | Protein function^c^ |
| *trm112* | 0.120 ± 0.02 | 15.2 ± 2.1 | 129.3 ± 23.4 | 0.093 ± 0.01 | 36.3 ± 9.1 | 363.9 ± 118.5 | tRNA methyltransferase |
| *dss1* | 0.109 ± 0.02 | 13.9 ± 2.9 | 134.0 ± 44.3 | 0.109 ± 0.02 | 40.8 ± 10.4 | 466.2 ± 160.9 | mRNA export |
| *caf1* | 0.109 ± 0.02 | 13.1 ± 4.4 | 122.7 ± 26.7 | 0.106 ± 0.02 | 43.3 ± 10.2 | 417.1 ± 111.7 | mRNA decay |
| *mlo3* | 0.102 ± 0.02 | 13.3 ± 3.4 | 130.6 ± 32.0 | 0.095 ± 0.01 | 40.3 ± 9.7 | 441.0 ± 143.3 | mRNA export |
| *crf1* | 0.099 ± 0.02 | 13.4 ± 2.7 | 134.9 ± 31.4 | 0.094 ± 0.01 | 36.7 ± 6.6 | 396.2 ± 84.5 | Transcriptional regulation |
| *ypa2* | 0.098 ± 0.02 | 9.5 ± 2.1 | 98.0 ± 22.7 | 0.085 ± 0.02 | 30.6 ± 5.9 | 370.1 ± 93.8 | Signal transduction |
| *dcd1* | 0.098 ± 0.02 | 13.7 ± 3.2 | 142.9 ± 38.1 | 0.081 ± 0.01 | 34.1 ± 6.2 | 434.2 ± 106.4 | DNA replication |
| *yox1* | 0.098 ± 0.02 | 14.1 ± 2.1 | 146.2 ± 27.4 | N.E. ^d^ | N.E. ^d^ | N.E. ^d^ | Transcriptional regulation |
| *cut8* | 0.096 ± 0.01 | 12.1 ± 2.1 | 128.0 ± 29.4 | 0.098 ± 0.01 | 51.0 ± 11.9 | 534.1 ± 174.6 | Proteolysis |
| *spo7* | 0.094 ± 0.02 | 14.0 ± 2.5 | 151.9 ± 31.0 | 0.091 ± 0.02 | 43.8 ± 7.8 | 494.9 ± 94.7 | Lipid metabolism |
| *nem1* | 0.095 ± 0.02 | 12.5 ± 3.2 | 132.5 ± 32.4 | 0.090 ± 0.02 | 42.8 ± 11.8 | 478.8 ± 91.7 | Lipid metabolism |
| *pvg5* | 0.095 ± 0.01 | 12.5 ± 1.9 | 136.2 ± 36.4 | 0.073 ± 0.01 | 33.6 ± 7.1 | 466.0 ± 103.4 | Cell wall biogenesis |
| *exo70* | 0.094 ± 0.01 | 16.7 ± 3.6 | 181.1 ± 46.5 | 0.075 ± 0.02 | 37.9 ± 11.6 | 531.6 ± 200.4 | Exocytosis |
| *upf3* | 0.094 ± 0.02 | 13.3 ± 2.3 | 149.1 ± 41.5 | 0.072 ± 0.01 | 36.9 ± 8.9 | 520.3 ± 130.7 | mRNA catabolic process |
| WT | 0.081 ± 0.01 | 11.3 ± 2.0 | 141.6 ± 31.1 | 0.075 ± 0.01 | 34.7 ± 6.0 | 468.9 ± 105.2 |  |

**S1 Table. Nuclear size mutants identified in genome wide screen in fission yeast**

^a, b^ Average value ± standard deviation (SD), n ≥ 30.

^c^ Description of protein function obtained from PomBase (http://www.pombase.org).

^d^ Not examined, synthetic lethal.
