# Supplementary material for: A systematic genomic screen implicates nucleocytoplasmic transport and membrane growth in nuclear size control
Source: PLoS Genet. 2017 May 18;13(5):e1006767. doi: 10.1371/journal.pgen.1006767 (PMC5436639; doi:10.1371/journal.pgen.1006767)
Supplement: S2 Table — (DOCX) [file pgen.1006767.s004.docx]

**S2 Table. N/C ratio of indicated strains**

| Strain | N/C ratio^a^ | Nuclear  volume^b^  (µm^3^) | Cell  volume^b^  (µm^3^) |
| --- | --- | --- | --- |
| WT (25°C) | 0.083 ± 0.008 | 10.0 ± 1.8 | 120.8 ± 23.7 |
| WT (36°C) | 0.084 ± 0.008 | 12.3 ± 2.0 | 146.9 ± 26.3 |
| WT + thiolutin (36°C) | 0.082 ± 0.015 | 12.4 ± 2.9 | 140.3 ± 35.8 |
| WT + CYH (36°C) | 0.128 ± 0.011 | 10.9 ± 2.1 | 123.8 ± 23.1 |
| *rae1-167* (25°C) | 0.080 ± 0.006 | 12.8 ± 2.9 | 159.8 ± 36.0 |
| *rae1-167* (36°C) | 0.128 ± 0.011 | 23.8 ± 4.0 | 187.1 ± 33.2 |
| *rae1-167* + thiolutin (36°C) | 0.080 ± 0.012 | 11.4 ± 1.8 | 144.9 ± 27.4 |
| *rae1-167* + CYH (36°C) | 0.092 ± 0.012 | 14.3 ± 2.8 | 142.5 ± 31.6 |
| *rae1-167pabpΔ* (36°C) | 0.126 ± 0.012 | 25.0 ± 4.9 | 199.7 ± 44.0 |
| *rae1-167atf1Δ* (36°C) | 0.122 ± 0.017 | 18.6 ± 6.5 | 155.8 ± 23.7 |
| *rae1-167mei4Δ* (36°C) | 0.126 ± 0.016 | 20.1 ± 2.8 | 162.8 ± 34.5 |
| WT (25°C) | 0.082 ± 0.009 | 10.0 ± 2.0 | 123.1 ± 28.7 |
| WT (36°C) | 0.084 ± 0.010 | 10.8 ± 1.3 | 130.1 ± 20.8 |
| *nem1Δ* (25°C) | 0.095 ± 0.019 | 12.4 ± 3.2 | 132.5 ± 32.4 |
| *nem1Δ* (36°C) | 0.101 ± 0.017 | 16.7 ± 3.3 | 166.3 ± 35.5 |
| *cut6-621* (25°C) | 0.081 ± 0.014 | 9.8 ± 2.4 | 123.0 ± 35.5 |
| *cut6-621* (36°C) | 0.085 ± 0.015 | 9.1 ± 2.3 | 108.1 ± 23.5 |
| *nem1Δcut6-621* (25°C) | 0.081 ± 0.011 | 9.9 ± 1.7 | 123.1 ± 20.1 |
| *nem1Δcut6-621* (36°C) | 0.082 ± 0.014 | 10.2 ± 2.1 | 127.0 ± 28.9 |
| *rae1-167* (25°C) | 0.080 ± 0.010 | 11.3 ± 2.7 | 140.5 ± 29.0 |
| *rae1-167* (36°C) | 0.123 ± 0.016 | 23.7 ± 5.7 | 192.6 ± 43.0 |
| *rae1-167cut6-621* (25°C) | 0.075 ± 0.011 | 10.3 ± 1.7 | 139.7 ± 30.0 |
| *rae1-167cut6-621* (36°C) | 0.098 ± 0.018 | 14.6 ± 4.1 | 150.3 ± 40.0 |
| *rae1-167nem1Δ* (25°C) | 0.098 ± 0.023 | 13.0 ± 3.6 | 133.4 ± 25.4 |
| *rae1-167nem1Δ* (36°C) | 0.136 ± 0.017 | 27.2 ± 5.7 | 200.5 ± 43.4 |

^a, b^ Average value ± standard deviation (SD), n ≥ 30.
