# Supplementary material for: A systematic genomic screen implicates nucleocytoplasmic transport and membrane growth in nuclear size control
Source: PLoS Genet. 2017 May 18;13(5):e1006767. doi: 10.1371/journal.pgen.1006767 (PMC5436639; doi:10.1371/journal.pgen.1006767)
Supplement: S3 Table — (DOCX) [file pgen.1006767.s005.docx]

**S3 Table. Nucleus-localised proteins are enriched and cytosol-localised proteins depleted in nuclear enriched samples**

| Strain | 2D enrichment analysis of average log_2_ nuclear enriched/whole cell ratios^a^ | | |
| --- | --- | --- | --- |
|  | Annotation category^b^ | 2D enrichment score | P value |
| WT (36°C) | Nucleus-localised | 0.12 | 0.017 |
|  | Cytosol-localised | - 0.11 | 0.005 |
| *rae1-167* (36°C) | Nucleus-localised | 0.18 | 0.006 |
|  | Cytosol-localised | - 0.12 | 0.00002 |

^a^2D enrichment analysis [21] of average log_2_ nuclear enriched/whole cell ratios from SILAC comparison of nuclear enriched sample to whole cell sample to confirm nuclear enrichment by nuclear enrichment protocol (see supplemental experimental procedures)

^b^Nucleus-localised and cytosol-localised categories from *S. pombe* ORFeome localisation data [19]
