# Supplementary material for: A systematic genomic screen implicates nucleocytoplasmic transport and membrane growth in nuclear size control
Source: PLoS Genet. 2017 May 18;13(5):e1006767. doi: 10.1371/journal.pgen.1006767 (PMC5436639; doi:10.1371/journal.pgen.1006767)
Supplement: S6 Table — (DOCX) [file pgen.1006767.s008.docx]

**S6 Table. Strains used in this study**

| PN5160 | *h^?^ crf1∆::kan^r^ cut11^+^:GFP:ura4^+^ ura4-D18 leu1-32 ade6* |
| --- | --- |
| UKK26-4C | *h^?^ dcd1∆::kan^r^ cut11^+^:GFP:ura4^+^ ura4-D18 leu1-32 ade6* |
| UKK34-2D | *h^?^ pvg5∆::kan^r^ cut11^+^:GFP:ura4^+^ ura4-D18 leu1-32* |
| UKK39-6D | *h^?^ cut8∆::kan^r^ cut11^+^:GFP:ura4^+^ ura4-D18 leu1-32 ade6* |
| UKK55-5A | *h^?^ trm112∆::kan^r^ cut11^+^:GFP:ura4^+^ leu1 ura4-D18 ade6* |
| UKK431-1B | *h^?^ dss1∆::kan^r^ cut11^+^:GFP:ura4^+^ ura4-D18* |
| UKK388-3C | *h^+^ caf1∆::kan^r^ cut11^+^:GFP:ura4^+^ ura4-D18 leu1-32* |
| UKK123-1B | *h^?^ spo7∆::kan^r^ cut11^+^:GFP:ura4^+^ ura4-D18 leu1-32 ade6* |
| UKK342-1A | *h^-^ nem1∆::kan^r^ cut11^+^:GFP:ura4^+^ ura4-D18 leu1-32 ade6* |
| UKK1045-4C | *h^+^ cdc25-22 cut11^+^:GFP:ura4^+^ ura4-D18 leu1-32* |
| UKK255-1D | *h^?^ cdc25-22 upf3∆::kan^r^ cut11^+^:GFP:ura4^+^ ura4-D18 leu1-32 ade6* |
| UKK330-1B | *h^?^ cdc25-22 exo70∆::kan^r^ cut11^+^:GFP:ura4^+^ ura4-D18* |
| UKK245-1B | *h^?^ cdc25-22 pvg5∆::kan^r^ cut11^+^:GFP:ura4^+^ ura4-D18 leu1-32* |
| UKK286-1C | *h^?^ cdc25-22 dcd1∆::kan^r^ cut11^+^:GFP:ura4^+?^ ura4-D18 leu1-32 ade6* |
| UKK324-1C | *h^?^ cdc25-22 ypa2∆::kanr cut11^+^:GFP:ura4^+^ ura4-D18 ade6* |
| UKK1511-1D | *h^?^ cdc25-22 nem1∆::kanr ade6? cut11:GFP:ura4+ ura4-D18 leu1-32* |
| UKK1512-1B | *h^?^ cdc25-22 spo7∆::kan^r^ cut11:GFP:ura4^+^ ura4-D18 leu1-32* |
| UKK1513-5B | *h^?^ cdc25-22 dss1∆::kan^r^ ade6 cut11^+^:GFP:ura4^+^ ura4-D18 leu1-32* |
| UKK1507-1C | *h^?^ cdc25-22 mlo3∆::kan^r^ ade6 cut11^+^:GFP:ura4^+^ ura4-D18 leu1-32* |
| UKK325-1A | *h^?^ cdc25-22 crf1∆::kan^r^ cut11^+^:GFP:ura4^+^ ura4-D18 ade6* |
| UKK244-5C | *h^?^ cdc25-22 trm112∆::kan^r^ cut11^+^:GFP:ura4^+^ ura4-D18 leu1-32* |
| UKK258-2A | *h^?^ cdc25-22 caf1∆::kan^r^ cut11^+^:GFP:ura4^+^ ura4-D18 leu1-32 ade6* |
| UKK268-2C | *h^?^ cdc25-2 cut∆8::kan^r^ cut11^+^:GFP:ura4^+^ ura4-D18 leu1-32 ade6* |
| PN1 | *h^-^* |
| PN10465 | *h^?^ rae1-167 ura4-D18 leu1-32 ade6* |
| PN5466 | *h^+^ car2∆::hph^r^ arg1-230 lys3-37 ura4-D18* |
| PN5467 | *h^?^ rae1-167 car2∆::hph^r^ arg1-230 lys3-37 ura4-D18* |
| UKK412 | *h^-^ rae1-167 cut11^+^:GFP:ura4^+^ ura4-D18 leu1-32 ade6* |
| UKK707-1A | *h^?^ rae1-167 pabp∆::kan^r^ cut11^+^:GFP:ura4^+^ ura4-D18 leu1-32 ade6* |
| UKK636-1A | *h^?^ rae1-167 atf1∆::kan^r^ cut11^+^:GFP:ura4^+^ ura4-D18 ade6* |
| UKK729-1B | *h^?^ rae1-167 mei4∆::kan^r^ cut11^+^:GFP:ura4^+^ ura4-D18 leu1-32 ade6* |
| UKK474-1C | *h^+^ cut6-621 cut11^+^:GFP:ura4^+^ ura4-D18 ade6* |
| UKK473-1C | *h^?^ cut6-621 nem1∆::kan^r^ cut11^+^:GFP:ura4^+^ ura4-D18 ade6* |
| UKK689-1A | *h^?^ rae1-167 cut6-621 cut11^+^:GFP:ura4^+^ ura4-D18 leu1-32* |
| UKK449-1A | *h^?^ rae1-167 nem1∆::kan^r^ cut11^+^:GFP:ura4^+^ ura4-D18 leu1-32 ade6* |
